# A global picture: therapeutic perspectives for COVID-19

**DOI:** 10.2217/imt-2021-0168

**Published:** 2022-02-21

**Authors:** Vivek P Chavda, Carron Kapadia, Shailvi Soni, Riddhi Prajapati, Subhash C Chauhan, Murali M Yallapu, Vasso Apostolopoulos

**Affiliations:** ^1^Department of Pharmaceutics & Pharmaceutical Technology, L.M. College of Pharmacy, Ahmedabad, Gujarat, 380009, India; ^2^Department of Pharmaceutics, K B Institute of Pharmaceutical Education & Research, Kadi Sarva Vishwavidhyalaya, Gandhinagar, Gujarat, 382023, India; ^3^Department of Immunology & Microbiology, School of Medicine, University of Texas Rio Grande Valley, McAllen, TX 78503, USA; ^4^South Texas Center of Excellence in Cancer Research, School of Medicine, University of Texas Rio Grande Valley, McAllen, TX 78503, USA; ^5^Institute for Health & Sport, Victoria University, Melbourne, VIC, 3030, Australia

**Keywords:** coronavirus, COVID-19, drug discovery process, drug repurposing, drug target, pandemic, SARS-CoV-2, vaccines

## Abstract

The COVID-19 pandemic is a lethal virus outbreak by severe acute respiratory syndrome coronavirus-2 (SARS-CoV-2), which has severely affected human lives and the global economy. The most vital part of the research and development of therapeutic agents is to design drug products to manage COVID-19 efficiently. Numerous attempts have been in place to determine the optimal drug dose and combination of drugs to treat the disease on a global scale. This article documents the information available on SARS-CoV-2 and its life cycle, which will aid in the development of the potential treatment options. A consolidated summary of several natural and repurposed drugs to manage COVID-19 is depicted with summary of current vaccine development. People with high age, comorbity and concomitant illnesses such as overweight, metabolic disorders, pulmonary disease, coronary heart disease, renal failure, fatty liver and neoplastic disorders are more prone to create serious COVID-19 and its consequences. This article also presents an overview of post-COVID-19 complications in patients.

## Plain language summary

Severe acute respiratory syndrome coronavirus-2 causes the coronavirus disease i.e., COVID-19. This article encompasses basic information about coronavirus and COVID-19. It includes information about viral transmission and subsequent events for COVID-19 in a person, and it presents different drugs and vaccines utilized for the management of COVID-19. Several natural drugs are proposed to manage COVID-19. It also has an overview about post-COVID-19 complications in recovered patients.

## Introduction

The coronavirus disease (COVID-19) was transmitted rapidly and extensively, and thus the World Health Organization (WHO) classified COVID-19 as a pandemic on 11 March 2020 [1,2]. The virus is characterized as a novel coronavirus (2019-nCoV) during the initial phase [3]. The viral genome is analyzed, and the International Committee for Taxonomy of Viruses listed it as ‘severe acute respiratory syndrome coronavirus-2' (SARS-CoV-2), as it had a genome identical to that of the coronavirus infection that caused the SARS outbreak in 2003 [4].

Even though initial cases were connected to the Huanan Seafood Market, where snakes, birds and other animals, including bats, were sold, the origin and source of SARS-CoV-2 are not clear [5]. In contrast to the transported cases, the majority of the initial patients worked at or were present at the supermarket, indicating that it was human-to-human or animal-to-animal transmission [6–8]. A probable source of this virus is the bat, as 96 % genomic sequence identification was illustrated between SARS-CoV-2 and the coronavirus bat genome CoV-RaTG13 that inhabited a region about 2000 kilometers from Wuhan. Pangolins have also been hinted at as a natural carrier-host for coronaviruses. A WHO delegation visited the Wuhan area on 22 January 2020 and confirmed human-to-human transfer [9]. The disease has expanded enormously across the globe since its inception 2 years ago, in December 2019, leading to more than 329 million cases of COVID-19, with over 5.5 million deaths thus far.

The SARS-CoV-2 test samples from various individuals have been isolated, and genomic sequences are made available immediately to researchers, in order to develop prophylactic and treatment options. As of February 2022, over 5 million SARS-CoV-2 RNA genomes had been shared through the global initiative on sharing all influenza data [10,11]. A number of nonspecific and broad-spectrum antivirals and anti-inflammatory medicines, supportive care and vaccines have been translated into human studies at unprecedented speeds [12,13]. Thus, patients suffering from critical COVID-19 infection are given antivirals (such as remdesivir), monoclonal antibodies (sotrovimab, bamlanivimab, etesevimab, asiriviamb, imdevimab), IL-6 and IL-1 inhibitors, mechanical ventilation and corticosteroids (inhalation, tablet or injection) [14,15]. Initial studies suggested that hydroxychloroquine, chloroquine and ivermectin were beneficial; however, many of the studies have been retracted as these drugs' benefits are controversial. In addition, convalescent plasma and interferons have been among the immunotherapies used to treat COVID-19 patients [16]. Immunoglobulins found in convalescent plasma can help patients recover from symptoms such as cough, pneumonia, and fever by inhibiting viral replication. Convalescent plasma can also help boost oxygen saturation and recovery, hasten the resolution of lung infiltration pathology and stabilize inflammatory mediators (by lowering CRP and IL-6), and it helps prevent leukocytosis and lymphopenia [17]. As a result, complications are reduced, hospital stays are shortened and mortality is decreased without causing adverse health effects. On the other hand, this type of immunotherapy is not very selective, and it has drawbacks, including short-term immunity and side effects such as allergic reactions, pulmonary edema and risk of disease transmission. As a result, more specialized therapeutic approaches are required. Monoclonal antibodies could be a better option with a higher specificity; however, determining specific targets is essential for monoclonal antibody production. Vaccination is another important strategy in managing the pandemic with mass vaccination roll-outs in most countries around the world [18,19].

This article encompasses the recent information regarding SARS-CoV-2 variants as well as the life cycle of the virus. It provides a general idea regarding therapeutic targets for the management of COVID-19 as well as drug repurposing for such targets for symptomatic relief. Monoclonal antibody treatments and vaccines are also discussed, along with herbal remedies and dietary supplements. Finally, post-COVID-19 complications and their management are presented. The keywords “COVID-19,” “vaccines,” “drug repurposing,” “monoclonal antibodies AND COVID-19,” “natural products AND COVID-19,” “post-COVID-19 complications” and “long COVID” were searched in PubMed, Scopus, Web of Science etc. and relevant articles were included in the article. Further, the search was refined to get specific information. Priority was given to the candidates under clinical development and confirmed from clinical trial registry number. However, for some candidates for which clinical trial data were not available, *in vivo* study data were considered for inclusion in this manuscript.

### Coronavirus strains

SARS-CoV-2 is an enclosed virus with an affinity RNA genomic structure, a single-stranded RNA of 29,891 bases that belongs to the *Corona viridae* family's beta subgroup [6,15]. The genome contains the genes for 29 proteins included in disease, replication and virion attachment to the host cells. The receptor-binding domain (RBD) of SARS-CoV-2 spike (S) protein interacts with human ACE2, increasing gene transcription and virus entry into host cells via endocytosis [4,20]. The RBD of the S protein is a crucial component of the coronavirus genome. According to physiological and functional analyses, the RBD from SARS-CoV-2 binds to ACE2 with a higher bonding capacity than RBD from other SARS-CoV viruses. Nevertheless, the high binding affinity could be due to human ACE2 protein variation [16].

**Figure 1.**
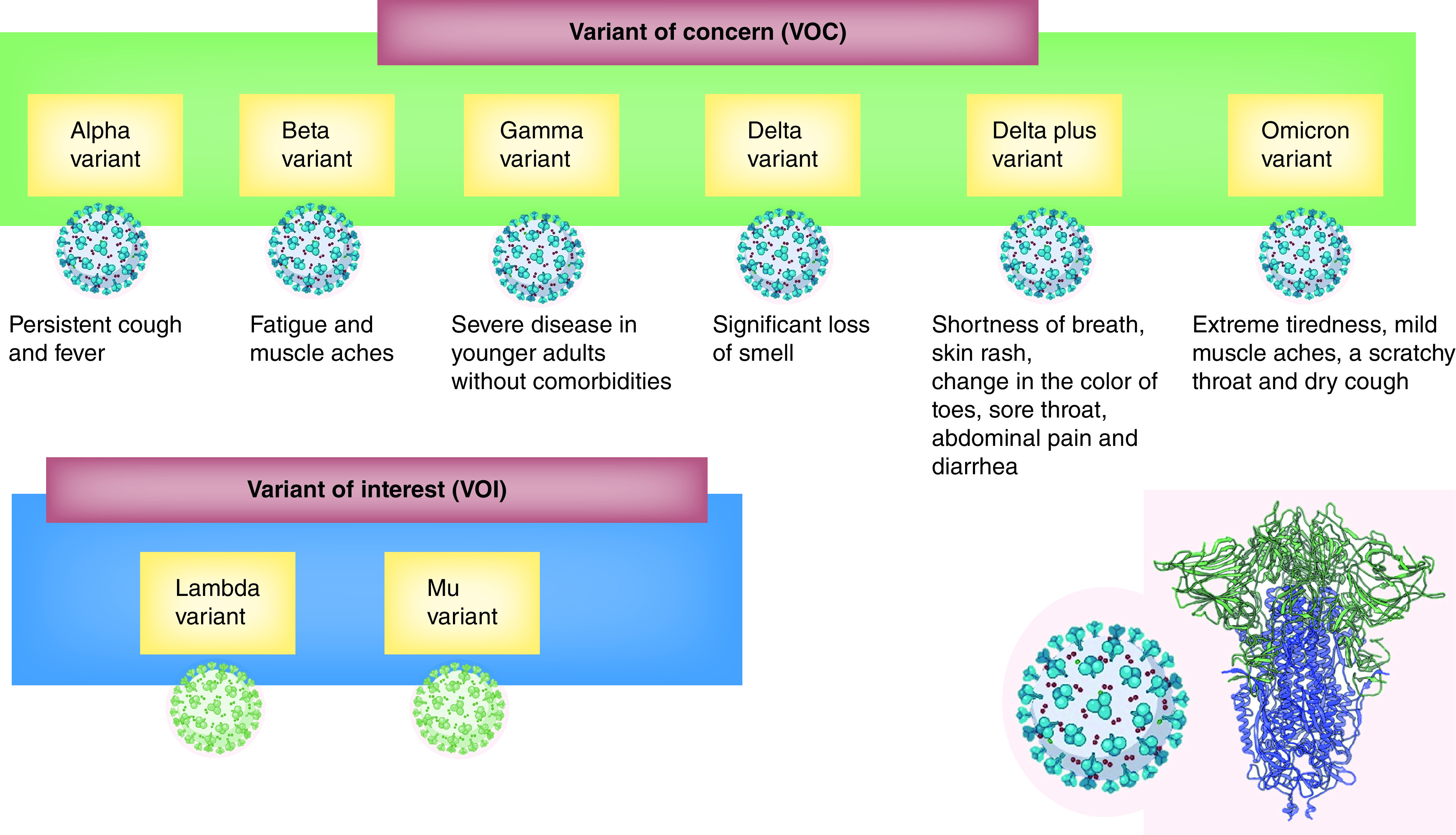
Different human variants of severe acute respiratory syndrome coronavirus-2.

**Figure 2.**
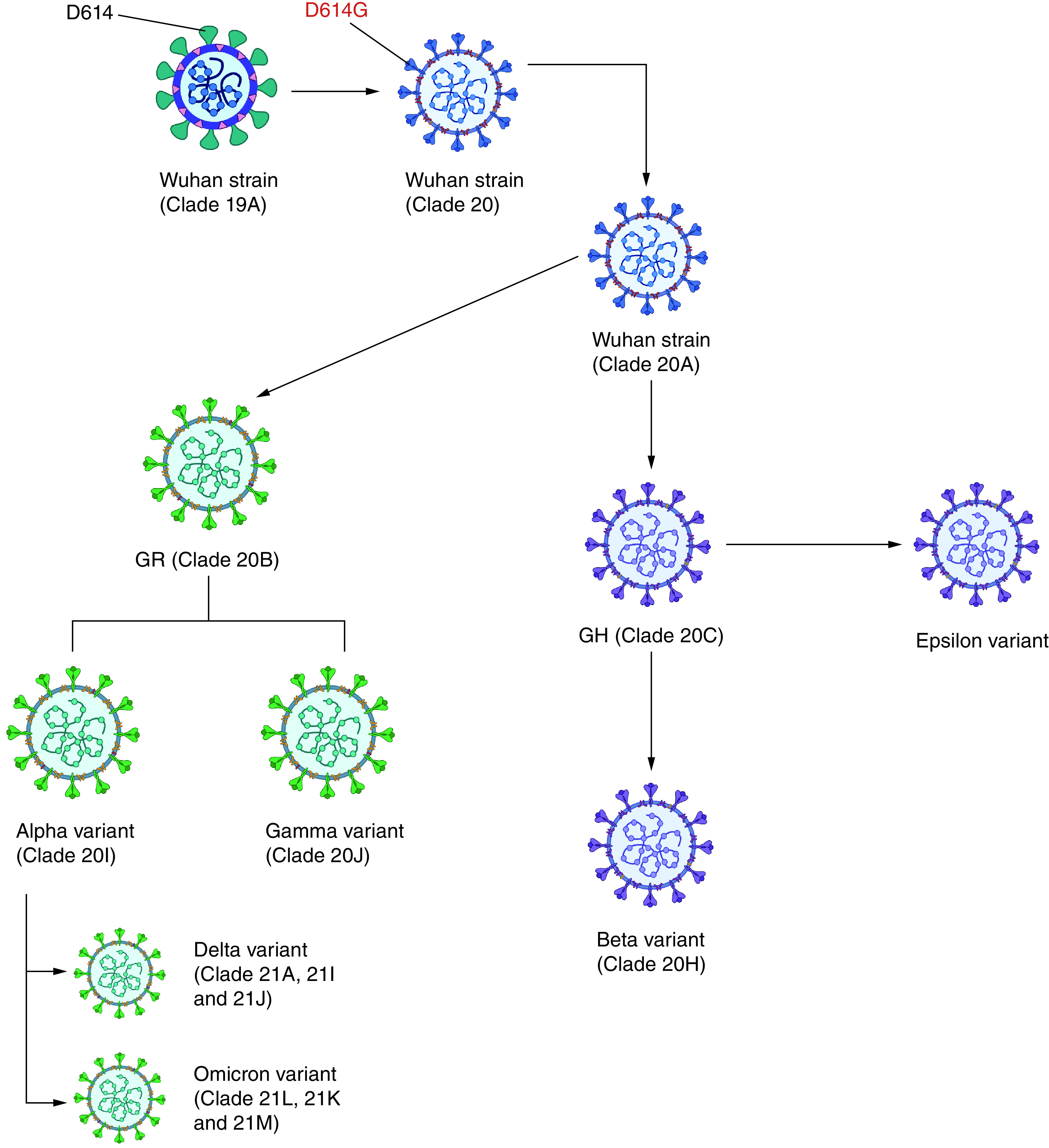
Phylogenetic tree of severe acute respiratory syndrome coronavirus-2. Created with Biorender.com.

Coronaviruses are prone to recombination and mutation as that of other RNA viruses. Mutations are unavoidable, although not all mutations are intentional or beneficial to the virus. The crowned protein of the coronavirus consists of 1273 amino acids divided into different regions [21]. Figure 1 documents various strains of the novel coronaviruses that infect humans [22]. Several mutant SARS-CoV-2 strains have been identified (Figure 2) [23]. The majority of the genetic mutations are insignificant and do not affect the virus's behavior. However, several mutations cause alterations in the S protein and other vital regions, leading to different viral characteristics. Any alteration between amino acid remnants 319–541 – particularly between 438 and 506 – might have a significant impact on the virus affinity to infections, transmissibility, severity and immunity-evading abilities [24].

Variants of concern (VOC) is a category used for variants of the virus where mutations in their spike protein receptor binding domain substantially increase binding affinity in RBD-hACE2 complex, while also being linked to rapid spread in human populations. One of the early mutants was identified in southeastern England in September 2020. That strain, now commonly known as B.1.1.7 (Alpha), rose rapidly to become the most common variant in the UK, with approximately 60% of new COVID-19 cases reported up until December 2020 [24]. The B.1.351 variant (Beta), first identified in South Africa, and the P.1 variant (Gamma), first identified in Brazil, spread to many countries across the globe. More recently, the Delta strain, first reported in India, spread globally at a fast rate, as it has a very high transmission rate and immune evasiveness [25]. A number of mutation sites include N501Y, where N asparagine is replaced with Y tyrosine at residue 501, resulting in higher transmissibility and mortality [11]. E484K (glutamic acid E substituted with lysine K) increases immune evasion and reinfection [26], and K417N (lysine K replaced with asparagine N) helps the virus avoid neutralizing antibodies, increasing the risk of reinfection [27]. Recently (26 November 2021), a new VOC of SARS-CoV-2 emerged in South Africa and was designated Omicron; it is from the B.1.1.529 lineage. Omicron has a larger number of mutations, which is concerning; it has an increased risk of reinfection; and it has quickly spread in many countries around the world. The Omicron variant has more than 50 mutations, of which 30 are associated with the viral S protein – a prime target for vaccine design [28,29].

Variants of interest (VOI) are specific genetic markers that are predicted to affect transmission, diagnostics, therapeutics, or immune escape. The mutation from the lineage C.37 is known as the Lambda variant, initially identified in Peru in August 2020, and it spread to over 30 countries. The Mu variant (lineage B.1.621) was first identified in Colombia, with outbreaks noted in other South American countries and Europe.

 The strain with E484Q and L452R mutations is known as a ‘double mutant’. Both of these point substitutions occur in the RBD's crucial receptor binding matrix (RBM) (amino acids 438–506) region. As a result, they may cause significant variation in the viral characteristics. According to a new analysis on the ‘California lineage’ (B.1.427/B.1.429), the L452R mutation (leucine L replaced by arginine [R] at position 452) may also have considerable immunity-evading capability [27]; this was designated Epsilon. The frequency of Epsilon rapidly decreased as it was outcompeted by the Alpha variant. Other VOCs include Zeta (P.2), Eta (B.1.525), Theta (P.3), Iota (B.1.526) and Kappa (B.1.617.1) that are currently not considered as VOCs.

## Life cycle of SARS-CoV-2

### Virus entry in to host cells

**Figure 3.**
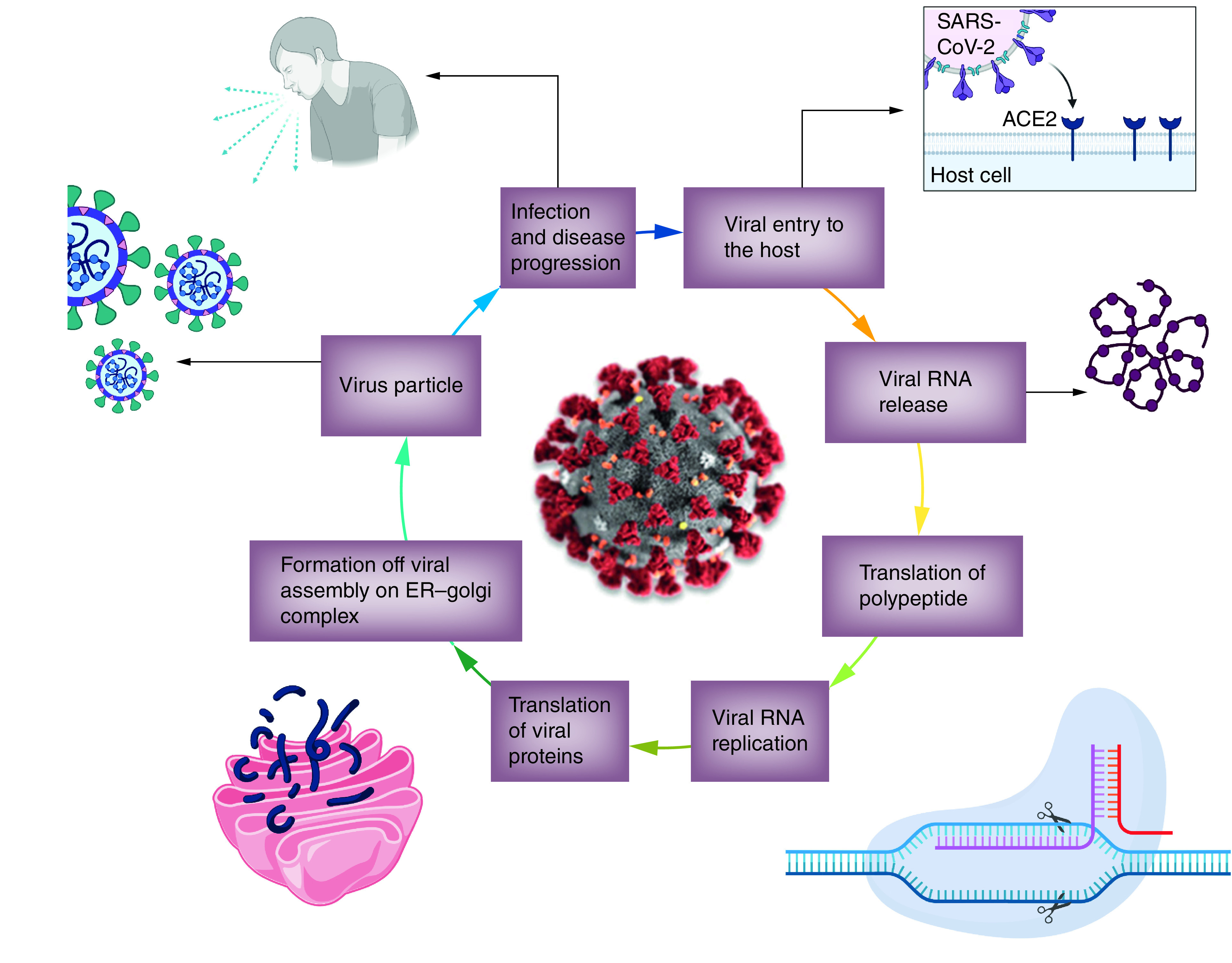
Life cycle of severe acute respiratory syndrome coronavirus-2 in the human body. Created with Biorender.com.

The SARS-CoV-2 enters the host cell through droplets produced by an infected person while sneezing or coughing. SARS-CoV-2 has the S glycoprotein on its surface, which has S1 and S2 functional subunits [30]. The surface exposed S1 subunit has 14–685 residues, and the S2 subunit contains 686–1273 residues; this two regions are responsible for receptor binding and membrane fusion, respectively. The S1 subunit comprises the N-terminal domain (14–305 residues) and RBD (319–541 residues), while the S2 subunit contains the fusion peptide (FP) (788–806 residues), heptapeptide repeat sequence 1 (HR1) (912–984 residues), HR2 (1163–1213 residues), tramsmembrane domain (1213–1237 residues) and cytoplasm domain (1237–1273 residues) responsible for viral fusion and entry [31]. HR1 and HR2 are essential for the viral fusion and entry function of the S2 subunit, and they form the six-helical bundle [32,33]. As the binding of the virus occurs at the ACE2 site, the entry of the virus into the host cell occurs through the formation of a proteolytic cleavage on the S protein of the virus by the host protease [34,35]. The binding and entry of the virus in the body are then fecilitated by several proteases expressed by host cells, including cathepsins L and B, trypsin, factor X, elastase, furin and TMPRSS2 [36]. Trypsin cleaves the viral S protein [37]. TMPRSS2 directly involves in the host interaction with the viral S protein [38]. The virus enters the host cell and unfolds, and the genome is first transcribed and then translated (Figure 3).

### Virus replication

**Figure 4.**
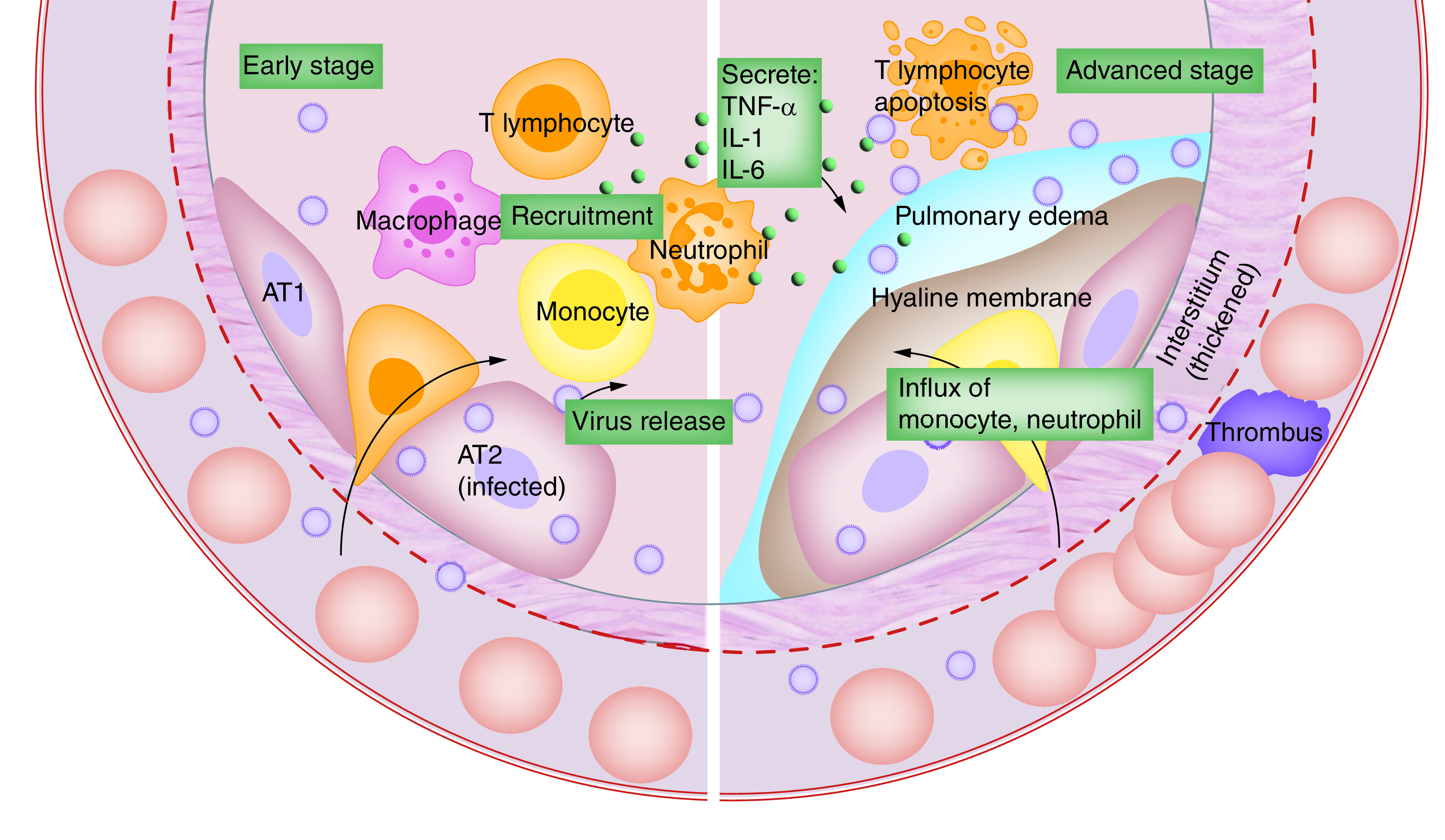
Immunopathogenesis of COVID-19 in early and advanced stages. Reused from [41] under CC BY 4.0 License.

The further step is the coronavirus genome's replication and transcription by continuous and discontinuous RNA synthesis at cytoplasmic membranes. The coronavirus has noticeable giant RNA genomes, each side 5′ and 3′ untranslated regions containing cis-acting secondary RNA structures, essential for RNA amalgam [39]. At the 5′ end, the genomic RNA contains two large open reading frames (ORFs; ORF1a and ORF1b) that occupy two-thirds of the capped and polyadenylated genome. As the virion releases the RNA within the cell, genomic RNA is transformed into pp1a and pp1ab and processed into individual nonstructural proteins (NSPS). Genetic translation forms the viral replication and transcription complex. According to NSPS expression, biogenesis of virus replication organelles occurs that contain characteristic perinuclear double-membrane vesicles, convoluted membranes and small open double-membrane spherules [40]. These elements help to create a protective microenvironment for viral genomic RNA replication. The transcription of subgenomic mRNAs contains the characteristic coronavirus mRNAs. Translated structural proteins translocate into the endoplasmic reticulum (ER) membranes and transit through the ER-to-Golgi intermediate compartment (ERGIC). After that, in the Golgi apparatus, newly produced genomic RNA results in the lumen of secretory vesicular compartments. Finally, virions are secreted from the infected cell by exocytosis, starting the virus life cycle all over again to nearby host cells [33,39]. The immunopathogenesis of COVID-19 is summarized in Figure 4; for more information about it, readers can refer to the work of Wang and co-workers [40].

## Therapeutic targets of COVID-19 & drug repurposing

**Figure 5.**
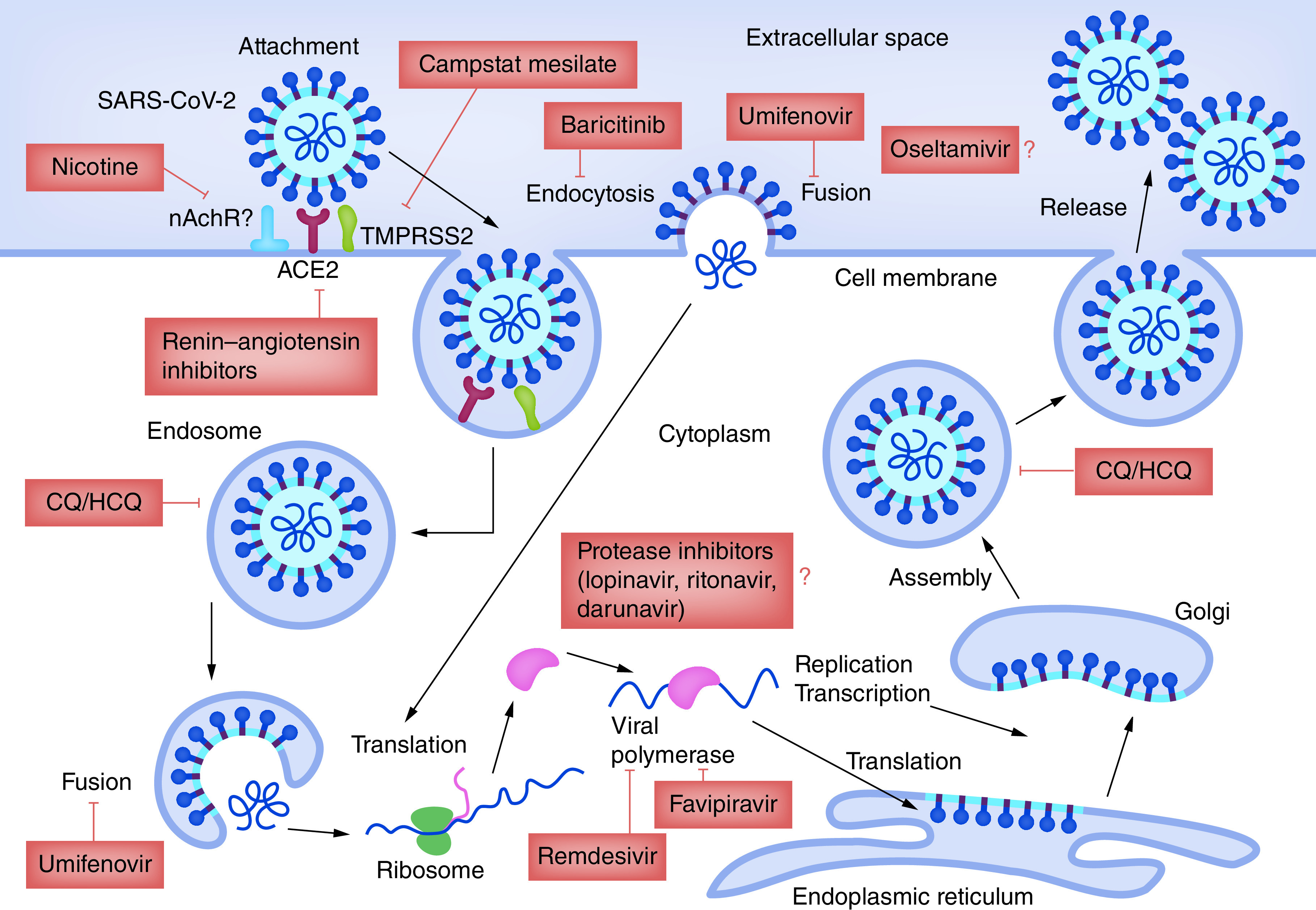
Coronavirus life cycle and target sites of potential antiviral agents. Reused with permission [44].

In the last year, a number of therapeutics have been repurposed for SARS-CoV-2, directed against different phases of the virus life cycle, from blocking its entry into host cells to inhibiting viral replication (Supplementary Table 1 [51,52,53,54,55,56,57,58,59,60,61,62,63,64,65] & Figure 5). The most challenging problem in developing treatments for coronaviruses is the immune response induced by coronaviruses that cannot be mitigated by direct antiviral drugs. Drug repurposing, also known as repositioning, is a fascinating platform in pharmaceutical research that recognizes novel therapeutic opportunities for future drugs such as corticosteroids, RNA-dependent RNA polymerase inhibitors, interferons, protease inhibitors, melatonin and teicoplanin [42]. Examples of repurposed drugs are the antimalarial drug hydroxychloroquine, utilized for the management of COVID-19 in a number of clinical trials and the antivirals remdesivir and favipiravir [43].

More recently, a drug that has the potential to be beneficial against SARS-CoV-2 is 2-deoxy-D-glucose, as it inhibits the glycolysis required for viral replication [45]. The Institute of Nuclear Medicine and Allied Sciences together with Dr Reddy's laboratory in Hyderabad, India, recently developed a 2-deoxy-D-glucose oral powder, which was approved in India for emergency use as an adjunct therapy in patients with moderate to severe COVID-19 [46,47]. The discovery of novel drugs and drug targets is ongoing. Thus, biomolecular targets, the obstruction of coronavirus structural protein, the targeting of viral enzyme, dipeptidyl peptidase 4 and membrane fusion blockers (ACE 2 and CD147 inhibitor) are primary areas for COVID-19 infection management [48]. The potential impact of biologic drugs on COVID-19 monitoring is enticing and contains cytokines, nucleic acid-based treatments targeting virus gene expression, bioengineered and vectored antibodies and different kinds of vaccines. Numerous drugs are presently being repurposed based on a fundamental understanding of viral pathogenesis and drug pharmacodynamics, as well as computational methods. In the present situation, drug repositioning could be viewed as a new treatment option for COVID-19. Drug repurposing is based primarily on two ideas. The first is that a single drug interferes with different targets, laying the groundwork for the discovery of new target pathways that lead to the known substance. The other idea is that targets related to disease are frequently pertinent to a variety of biological processes of pathogenesis, paving the way for the classification of a new indication for the established target. In theory, a drug that acts on such basic traits could be useful for a variety of disorders [42]. Recent research described interdisciplinary network-based systems pharmacology, a method that measures the interconnection between the coronavirus–human cell network interface and drug targets in the human protein–protein engagement network, allowing for the efficient detection of SARS-CoV-2 repurposed drugs. Using this strategy, research was able to recognize 30 prospective repurposed drugs against COVID-19 [49,50].

### Drug repurposing

Drug repurposing (also called reprofiling, retasking and medication rescue as well as repositioning) is a method that identifies existing drugs against one disease to be used for another disease [66]. This method is thought to be a cost-effective, quick and reliable solution. To develop new drugs takes a long time, at least 10–15 years from discovery to approval, costing more than $1 billion, with a success rate of just 2%. Thus, repurposing developed drugs for the treatment of multiple diseases has become a common approach, since it utilizes de-risked substances along with well-known pre-clinical, pharmacokinetic and pharmacodynamic profiles that would be fast tracked through to phase 3 human clinical trials, making the drug discovery procedure comparatively inexpensive and quick. As a result, the WHO as well as other health organizations have reconsidered the effectiveness of approved and potential medications to treat existing health problems (Supplementary Table 2 [75,76,77,78,79,80,81,82,83,84,85,86,87,88,89,90,91,92,93]) [67]. On the downside, however, due to the 'burden' of intellectual property and the related expenditures, many pharmaceutical companies are cautious about fully exploiting the possibilities of drug repurposing. Drug repurposing strategies, on the other hand, can be split into two major categories: bioinformatic techniques and experimental data. Analytical methods of drug repurposing are often data-driven and include a thorough evaluation of gene expression, chemical structure, proteomic data, computational chemistry techniques (including molecular docking and dynamics), pathway or network mapping and retrospective analysis using the electronic health records of approved drugs [68–74].

**Figure 6.**
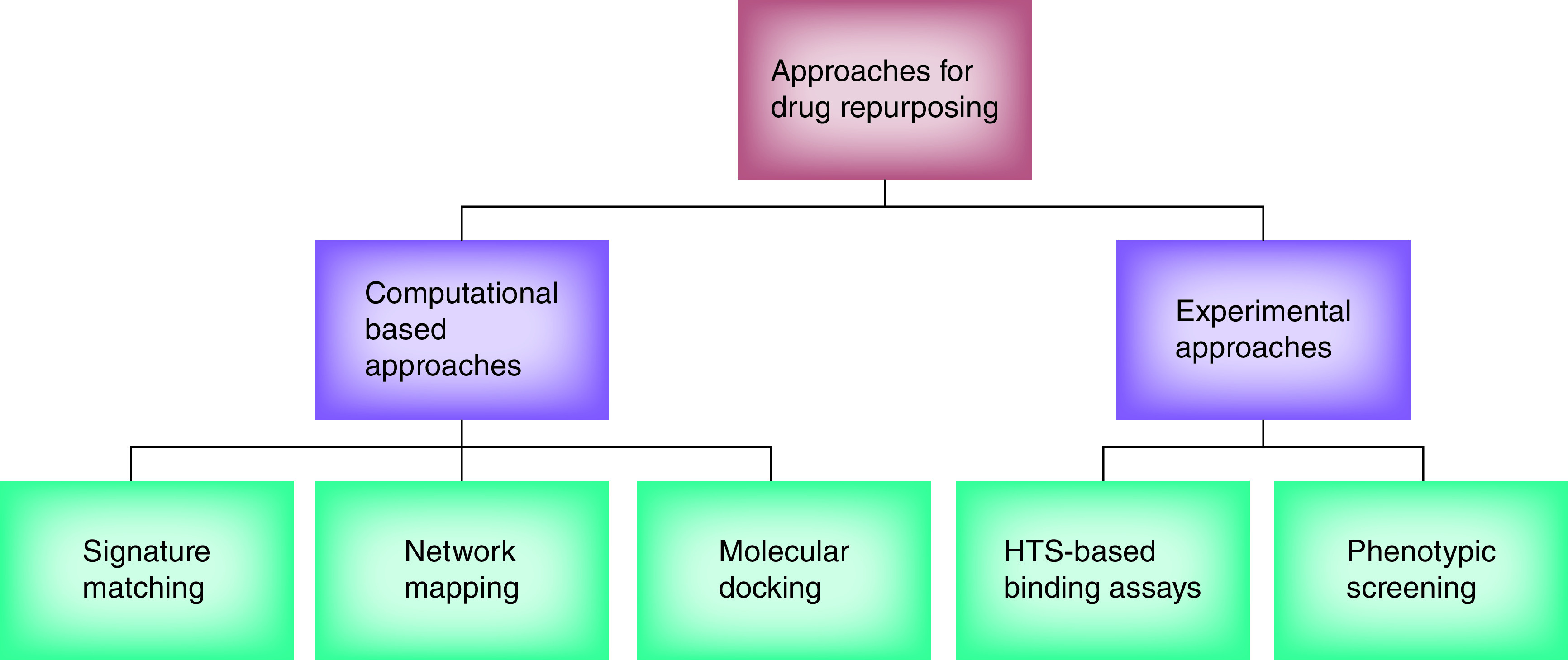
Approaches to drug repurposing.

According to Yan Ling NG and colleagues, *“Computational approaches make use of machine learning and algorithms to model disease and drug interaction, while experimental approaches involve* *a more traditional wet-lab experiments”* [94]. COVID-19 drug repurposing, like all drug repurposing developments, needs to go through three stages before it can be perceived for advancement through the product portfolio: the recognition of drug candidates, the mechanistic evaluation of drug effects in pre-clinical models and the assessment of the drug's effectiveness in phase II clinical trials [95]. Unique relationships between the repurposed drug and COVID-19, as well as distinctions in specific populations or dosing routines that may have serious implications, should be looked into [94]. The approaches to drug repurposing (Figure 6) are as follows.

#### ‘Signature’ matching

Each medication or active pharmaceutical candidate has specific features, or a ‘signature’, such that its transcriptomic, structural or adverse side effects are known and can be repurposed by correlating these signatures with previously known diseases or drugs [72]. Thus, drug–disease, drug–gene or drug–drug comparisons are analyzed and drugs are repurposed for other uses. A number of drugs have been repurposed, including an anti-epileptic drug [74].

#### Computational molecular docking

The binding effectiveness is assumed for the drug and the target site entity by using structure-based computational methods [96]. Using computational chemistry, large- and small-scale screens against a disease target can be carried out with known drugs. However, this approach has its own disadvantages, such as the 3D structure for multiple targets is not explained or there is a deficiency of macromolecular screenable database, which can give structural data for a wide range of molecular classes of compounds [97].

#### Network mapping

One of the most prevalent medication repurposing strategies is molecular pathway or network mapping. Since many established drug targets are not drug-driven and direct inhibition can induce serious side effects, network mapping can alert upstream or downstream drug targets, allowing for drug repurposing [98]. Through network mapping approaches, medication, and disease connections can be created depending on the expression of genomic patterns and disease pathophysiology. Such maps and links offer new opportunities for drug repurposing [99].

### Artificial intelligence & drug repurposing

Drug repurposing approaches and research are being revolutionized by advancements in information systems for artificial intelligence (AI) and ‘big-data analytics’. Computational methods that can anticipate novel drug target involvement with considerably higher reliability than traditional approaches can be built with the assistance of machine learning techniques. When paired with disease characteristics and treatment alternatives, massive amounts of information created by high-throughput next-generation sequencing from several infected individuals can lead to the discovery of novel infection biomarkers and therapeutic targets [100]. AI-operated with multi-task learning based machine-learning algorithms are used to enhance the pharmacological response induced by the engagement of numerous pharmacological targets [101]. The effects of this approach can be appreciated in a recent study that used a statistical strategy for analyzing diversified information from initially recorded drug target interrelations to assume novel interactivity with even higher precision, the effect of this technology. The technique, called deepDTnet, combines complexes linking various drugs with drug target sites and infection databases with the use of deep learning (a type of machine learning) [102]. Drug repurposing approaches might also be utilized to identify medications that could be used as antiviral agents. Drugs or compounds with antiviral activity may be discovered by searching a library of small molecule compounds against viral medications using computational approaches [103,104]. Vaxine Pty Ltd, a biotechnology company located in South Australia, uses computational and AI-based techniques to speed up widespread infection vaccine and drug manufacturing, with the goal of decreasing drug development time from decades to weeks [105].

## Vaccines for COVID-19

**Figure 7.**
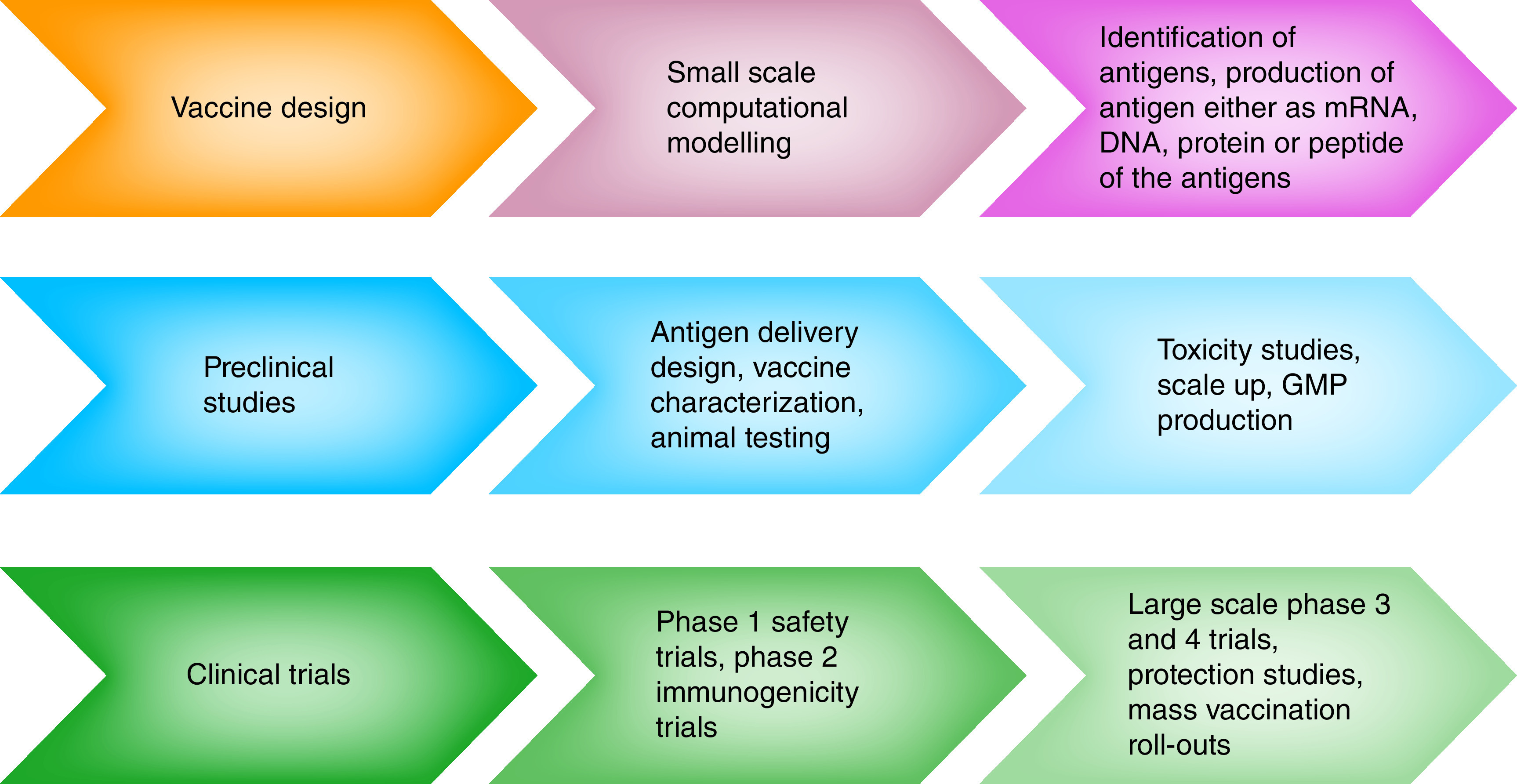
Various phases of vaccine development.

Enormous efforts have been focused on designing anti-SARS-CoV-2 vaccines with the primary aim of eliciting antibody responses against the virus, which would block and neutralize it once it has infected the host. According to WHO statistics as of 11 February 2022, a total of 194 vaccines are undergoing pre-clinical testing and 139 candidates have entered human clinical trials at unprecedented speed (Supplementary Table 3 [133,134,135,136]); currently, there are 10 WHO-approved vaccines [82,106,107]. A number of vaccine formulations, including inactivated vaccines, nucleic acid vaccines, viral vector-based vaccines and protein subunit vaccines, have been developed, with or without adjuvants, with the aim of eliciting appropriate immune responses and protection against SARS-CoV-2 infection [108]. Figure 7 shows the various phases of vaccine development. More than 9.7 billion people are vaccinated, which accounts for 76% of the population [109]. Well over 238 million additional doses have been injected globally, with several nations, especially lower-income countries, expected to start soon. There are 24 vaccine candidates with emergency use authorization (EUA), and 139 vaccine candidates are in clinical development using different vaccine platforms. Booster doses were initially recommended for those individuals with low vaccine efficacy or for those who are immunocompromised [110]. However, more recently, a booster doses have been initiated for those who received the vaccine more than 6 months prior, with priority to those above 60 years of age. The propagation of such viral pathogens in the community leads to mutations that alter the viruses' pathogenesis, infectivity and invasiveness, or a mixture of these [111]. Despite the fact that the Alpha variant (B.1.1.7) has several mutations on the S protein, the most significant of which is the N501Y mutation, recent studies have shown that sera obtained from people immunized with mRNA-1273 or BNT162b2 had similar neutralizing functions against the B.1.1.7 variant when compared with the wild-type form [111,112]. According to Bernal and colleagues, *“Effectiveness after one dose of vaccine (BNT162b2 or ChAdOx1 nCoV-19) was notably lower among persons with the delta variant (30.7%; 95% confidence interval [CI], 25.2 to 35.7) than among those with the alpha variant (48.7%; 95% CI, 45.5 to 51.7); the results were similar for both vaccines. With the BNT162b2 vaccine, the effectiveness of two doses was 93.7% (95% CI, 91.6 to 95.3) among persons with the alpha variant and 88.0% (95% CI, 85.3 to 90.1) among those with the delta variant. With the ChAdOx1 nCoV-19 vaccine, the effectiveness of two doses was 74.5% (95% CI, 68.4 to 79.4) among persons with the alpha variant and 67.0% (95% CI, 61.3 to 71.8) among those with the delta variant”* [113]. The Cov-Boost experiment investigated the use of seven different vaccines as boosters following two doses of either the AstraZeneca or Pfizer vaccine: AstraZeneca, Curevac, Johnson and Johnson (Janssen), Moderna, Novavax, Pfizer, and Valneva. The trial discovered that all vaccinations (except Curevac, which was discontinued) increased immunological response; however, the quantity of antibodies varied greatly depending on the vaccine combination [114–116].

### Inactivated (killed) vaccines

Inactivated or killed vaccines contain an inactive form of the virus, being inactivated by physical or chemical methods in order to destroy infectivity yet retain immunogenicity. Such inactivated vaccines have been approved for human use against polio (Salk vaccine), hepatitis A, rabies, typhoid, cholera, plague, pertussis and most influenzas. Inactivated vaccines are effective, as the whole virus is used and there are multiple epitopes by which immune responses are generated. Thus far, seven vaccines are being tested for SARS-CoV-2 [117]. Covaxin is India's first COVID-19 vaccine developed between Bharat Biotech and the Indian Council of Medical Research (ICMR)–National Institute of Virology (NIV). Covaxin is administered in two doses, 28 days apart, and is effective against the Indian (B.1.617), Brazil (P1) and UK (B.1.1.7) variants [118]. Eighty million doses of Covaxin were produced in the October 2021 and the production capacity is to be increased to 700–800 million doses per annum by March 2022. Two other inactivated vaccines approved for use are Chumkov Center (KoviVac) vaccine, which is approved in Russia [119], and RIBSP QazCovid-in vaccine, which is approved in Kazakhstan [120]. Another is Minhai Biotechnology Co SARS-Cov-2 vaccine (Vero Cells, KCONVAC, KconecaVac), which is approved in China and Indonesia [121]. Sinopharm (Beijing) is approved in 41 countries with clinical trials in seven countries, including Argentina, Peru and the United Arab Emirates [122]; Sinopharm (Wuhan) is approved in China for emergency use [123] and Sinovac (CoroVac) vaccine has been approved and tested in 25 countries (Supplementary Table 2 [75,76,77,78,79,80,81,82,83,84,85,86,87,88,89,90,91,92,93]) [124].

### Protein subunit vaccines

Protein subunit vaccines contain part of the viral protein mixed with an appropriate adjuvant or conjugated to immunogenic carriers [125]. The major limitations of these vaccines are that only a few components from the virus are used, which may not be representative of the complexity of the entire structure of the virus and hence their protective ability may be limited [108]. However, such vaccines do not contain any live virus components, making them much safer than inactivated (killed) vaccines. Initial clinical results from studies of the NVX-CoV2373 vaccine (Novavax), a recombinant nanoparticle vaccine against SARS-CoV-2 that encompasses the newly designed strain's full-length S glycoprotein plus Matrix-M adjuvant, had shown that the immunization was safe and connected with a sturdy immune reaction in healthy adult participants [126]. 21 vaccines are in phase 3 human clinical trials based on this vaccine platform. Anhui Zhifei Longcom (RBD-dimer) is approved in China and Uzbekistan while FBRI EpiVac Corona, is approved in Russia and Turkmenistan (Supplementary Table 6 [190,191,192,193,194,195,196]) [106]. Currently, there are six protein subunit vaccines in the market under emergency use approval in different parts of the globe.

### Viral vector vaccines

In the case of vector vaccines, a safe virus such as adenovirus or poxvirus is utilized to deliver the gene encoding for proteins from SARS-CoV-2 into the cells (mostly S protein), initiating immune responses [127]. After a brief pause due to recent adverse event reports of thrombosis with thrombocytopenia syndrome, the CDC and US FDA approved the emergency use of the Johnson and Johnson vaccine [128]. However, a thorough examination of the data revealed that the vaccine's potential benefits outweighed the risks. This vaccine is a single-dose vaccine. Another vaccine in this class is Covishield, developed by Oxford University and AstraZeneca, and various countries around the world are producing it. It is being manufactured by the Serum Institute of India. Covishield is a viral vector vaccine that is administered in two doses, 3 months apart, showing antibody responses, and it is effective against the Indian Delta variant (B.1.617) but generates lower antibody titers [129]. Other approved vaccines include CanSino Ad5-nCoV, approved in five countries, and Gamaleya (Sputnik V), developed by Russia, which is effective against the Brazilian (P.1) and South African (B.1.351) mutants [130]. Bharat Biotech has developed a nasal vaccine, BBV154, for localized IgA antibody responses and protection at the entry point of the virus; other vaccines act by generating IgG systemic responses (Supplementary Table 3 [133,134,135,136]) [19].

### mRNA-based vaccines

mRNA-based vaccines work by delivering the region of interest (i.e., S protein) as RNA into the cytoplasm of antigen presenting cells and stimulate the immune response by expression of viral protein using cell machinery of antigen presenting cells [127]. The Pfizer BioNTech and Moderna vaccines have been approved for emergency use. The Indian strain (B.1.617) and the UK strain are both sensitive to Pfizer BioNTech (B.1.1.7) [131]. A recent publication by *Nature* reports the use of combination vaccine in which the first dose of AstraZeneca vaccine is administered and the second dose is administered as the Pfizer vaccine [132]. The study involved 600 participants who had received the first dose of AstraZeneca vaccine, with half receiving a second dose of Pfizer and the other half receiving a second dose of AstraZeneca [132]. The results of this study reported that those who received the Pfizer vaccine as the second dose had seven-times higher neutralizing antibodies compared with the second dose being that of AstraZeneca [132]. This opens a whole new arena of these kinds of mix-and-match (or prime-boost) vaccines. Recently, the FDA fully approved the Pfizer BioNTech vaccine for COVID-19.

## Antiviral monoclonal antibodies as COVID-19 therapeutics

### Antibodies against the S region

Based on the active regions of SARS-CoV-2, antibodies against the S protein and other proteins have been generated and used as a treatment option for patients with COVID-19 [137]. The idea is that the antibodies injected into a COVID-19 patient bind and block SARS-CoV-2. In accordance with this approach, the Regeneron monoclonal antibody cocktail (casirivimab/imdevimab sold under the brand name REGN-COV2) was developed [138]. The monoclonal antibodies bind to the RBD of SARS-CoV-2, which is part of non-overlapping epitopes of the S protein, and hence inhibits the binding of the virus to the human ACE2 receptor [139]. A phase 1/2/3 study (NCT04426695) of hospitalized COVID-19 patients is currently being conducted to evaluate the effectiveness of REGN-COV2, while a phase 3 study (NCT04452318) is determining the prophylactic use of the cocktail. Another such product in development is Eli Lilly's LY-CoV555 cocktail, which targets different epitopes of the S protein [140], and on 7 October 2020, Eli Lilly declared that it had submitted an EUA request for monotherapy in patients who are at high risk, while the FDA authorized its emergency use on 9 November 2020. The FDA stated that *“it is reasonable to believe that bamlanivimab may be effective for the treatment of mild to moderate COVID-19 in adults and pediatric patients who are at high risk of progression to severe COVID-19 and/or hospitalization, and when the potential benefits outweigh the known and potential risks of such product*” [141].

### Antibodies against cytokines & chemokines

Another mechanism by which monoclonal antibodies can reduce the effects of SARS-CoV-2 is by blocking inflammatory cytokines such as IL-6. Monoclonal antibodies such as tocilizumab acts by blocking the IL-6 pathway and in turn prevents the inflammatory response [142]. In addition, blocking granulocyte-macrophage colony stimulating factor, which is responsible for a variety of inflammatory reactions, including lung inflammation, is another promising monoclonal antibody treatment [143]. Further, CC-receptor 5 expressed on the surface of cells is increased in the lungs of COVID-19 patients, and leronlimab, an anti-CCR5 monoclonal antibody approved for use in HIV-1 infection, shows promising results for the management of COVID-19 [144]. The REMAP-CA study, with 803 participants, evaluated tocilizumab in COVID-19 and showed good benefit [145]. According to the RECOVERY and REMAP-CAP study analysis, it tocilizumab could be used for the treatment of COVID-19-associated pneumonia and high C-reactive protein levels in patients with moderate to severe disease [146]. Examples of some monoclonal antibodies under review for the management of COVID-19 are summarized in Supplementary Table 4 [147,148,149,150,151,152,153,154,155,156,157,158,159].

## Natural compounds, dietary supplements & nutraceuticals against SARS-Cov-2 infection

It has been reported that several natural compounds and their derivatives were effective against SARS-CoV-2 infection (Supplementary Table 5 [147,148,166,167,168,169,170,171,172,173,174,175,176,177,178,179]) [160–163]. It has been documented that a number of herbal extracts belonging to the Polygonaceae family inhibit the interaction of viral S protein with host ACE2 [164], and one such example is emodin [165].

Two naturally occurring flavonoids, myricetin and scutellarein, have been validated as possible inhibitors of SARS-Cov-2 helicase NSP133 [180]; helicase is involved in viral genomic RNA replication, transcription and translation [181]. In addition, Rhizoma Cibotii; dried *Cibotium barometz* and dioscoreae rhizoma rhizomes; *Doscorea batatas* tubers; the flavonoids herbacetin, rhoifolin and pectolinarin [182]; and betulinic acid and savinin triterpenes show a substantial decrease in SARS-CoV 3CL protease activity [183]. The papain-like cysteine protease (PLpro) plays an important role in SARS-CoV-2 viral genomic RNA replication and has been used as a key drug target for the development of anti-SARS-CoV-2 drugs [184]. Moreover, *Ganoderma lucidum* extracts are effective against COVID-19 by targeting RNA-dependent SARS-CoV-2 RNA polymerase, an important enzyme useful for the synthesis of viral RNA [168,185]. Current data emphasize the importance of nutritional supplementation, and it may be advantageous in minimalizing viral load and hospitalization for COVID-19 patients if given in higher than acceptable dietary doses [186,187]. A lack of these vitamins and nutrients in serum concentrations may lead to a decrease in the immune system's fine performance, which is one of the components that contribute to a weak immune state. When represented by rigorous clinical studies, vitamin and micronutrient dietary supplements, on the other hand, provide a very convenient and simple approach with promising wider activity and lengthy health advantages (Supplementary Table 6 [190,191,192,193,194,195,196]). When the health benefit-to-risk ratio is considered, vitamins and micronutrients are likely valid with minimal risks. In contrast, the risk associated with new drugs as well as some vaccines is low. As a result, nutrient intake shows potential as a strategy against SARS-CoV-2 infection [188,189].

## Post-COVID-19 complications

Surviving COVID-19 may only be the beginning of many battles for some. Long-term impacts can be predicted using new information, as well as earlier learning with more serious respiratory infections and the larger post-intensive care syndrome, a combination of physical, cognitive and psychological disorders that can arise post-intensive care. Acute respiratory distress syndrome (ARDS) is common in COVID-19 individuals who develop severe symptoms, necessitating hospitalization [197]. ARDS can cause irreparable lung tissue damage, which can result in long-term breathing issues. Such individuals require mechanical breathing between 33% and 75% of the time, usually for weeks. Patients on ventilators are at higher risk of respiratory diseases that can result in serious consequences and a risk of chronic lung damage. Patients with post-intensive care syndrome have a greater rate of cognitive and physical impairment, which can last a long time [198]. COVID-19 diseases are also linked to a higher risk of extrapulmonary consequences, which can result in long-term illness, disability and mortality. Cardiovascular injury, acute ischemic or hemorrhagic stroke, neurological impairments, acute renal injury (requiring dialysis) and liver injury are among them. COVID-19 thromboembolic complications, such as pulmonary embolism, stroke and various microinfarctions, can result in a variety of long-term organ injuries. Critical pneumonia has been linked to an elevated chance of heart disease in both the initial stages of the diseases and subsequent years, irrespective of ARDS [199]. The higher number of cases underlying cardiovascular illness, hypertension and diabetes in patients with critical COVID-19 infection, as well as COVID-19 independent effects on the cardiovascular system, suggests that COVID-19 survivors will have a much higher risk of heart disease [199].

Even if COVID-19 patients recover physically, they are also at risk of long-term mental health issues. Long-term psychological distress and post-traumatic stress disorder affect more than half of patients who have had serious illness. Isolation and loneliness, job loss and financial hardship, intensified child care and familial responsibilities and worry about family or other connections developing COVID-19 could all worsen the mental health consequences of surviving the virus [197]. There are also a number of bacterial and fungal co-infections that can be related to pre-existing morbidity (diabetes, lung illness) or can arise as a hospital-acquired infection such as ventilator-related pneumonia. There have been a number of reports of patients with COVID-19 developing mucormycosis (fungal infection) during or after treatment [200,201].

## Conclusion & future perspective

Since the inception of SARS-CoV-2, numerous research efforts have made worldwide to manage the COVID-19 pandemic. Initially when no treatment options were available, researchers quickly switched gears to evaluate available drug moieties against SARS-CoV-2 as a part of drug repurposing. The range of therapeutic strategies approved to treat COVID-19 is expanding quickly and includes both FDA-approved drugs and drugs readily accessible under EUA. In the case of a pandemic with viral infection, mass vaccination seems to be the most effective means of COVID-19 eradication. As of November 2021, there were 24 vaccine candidates with different platforms under emergency use, with more than 154 vaccine candidates in different stages of clinical development. Mucosal vaccine candidates are also under development for generating localized immunity to halt the viral spread and gaining sufficient immune protection. Therapeutic interventions such as convalescent plasma therapy and the use of monoclonal antibodies are aimed at providing symptomatic relief and rapid recovery in mild to moderate COVID-19 cases. Furthermore, SARS-CoV-2 has shown many variants that bring new episodes of endemic globally. Moreover, post-COVID-19 complications have surfaced to further complicate the picture of COVID-19 therapeutic management. A number of treatment and prophylactic options have been developed in a short period of time, with many positive outcomes thus far. This unprecedented crisis requires the scientific community's exceptional efforts to tackle the problem effectively and avoid further losses of human life and health. The future is optimistic, but it will take several more years of combined, worldwide effort to completely manage the pandemic.

**Executive summary**

**Introduction**

- Severe acute respiratory syndrome coronavirus-2 (SARS-CoV-2) is responsible for the recent COVID-19 pandemic, with a number of variants being identified.
- Observations of viral infections that have the tendency to affect epidemics and are spread via the respiratory tract must not be dismissed.
- Lymphocytopenia and cytokine storm are two significant embodiments of an innate immunity to COVID-19.

**Therapeutics for COVID-19**

- A number of repurposed US FDA-approved drugs have been identified and tested in human clinical trials for the treatment of disease.
- Antiviral agents, inflammation inhibitors/antirheumatic drugs, low molecular weight heparins, plasma and hyperimmune immunoglobulins are the categories of drugs used, based on the pathophysiological features and different medical stages of COVID-19, especially in patients with moderate to severe COVID-19.
- Natural products have been shown to have anti-inflammatory and antiviral properties.
- Monoclonal antibody treatments are showing promise in patients with COVID-19.

**Vaccines for COVID-19**

- Vaccines have been fast tracked with new methodologies being approved for emergency use.
- Vaccines against COVID-19 are being developed using either the spike protein of severe acute respiratory syndrome coronavirus-2 or the entire virus.
- Immunization efficacy evaluated in randomized, controlled trials under optimal circumstances may vary slightly from immunization efficacy evaluated in randomized trials under non-ideal conditions and in diverse populations.

**Post-COVID-19 complications**

- Post-COVID-19 complications in some individuals is an ongoing issue.
- More investigation, time and education programs are needed to better understand and recognize post-COVID-19 complications in a range of cultures and environments.
- This unprecedented crisis requires the scientific community's exceptional efforts to tackle the problem effectively and avoid further loss of human life.
