# Supplementary material for: A global picture: therapeutic perspectives for COVID-19
Source: Immunotherapy. 2022 Feb 21:10.2217/imt-2021-0168. doi: 10.2217/imt-2021-0168 (PMC8884157; doi:10.2217/imt-2021-0168)
Supplement: Supplementary file 1 [file supplementary_table_1.docx]

**Supplementary Table 1:** Summary of potential therapeutic targets for COVID-19

| **Target** | **Examples** | **Mechanism of action** | **Pros** | **Clinical trial** | **Cons** | **Ref** |
| --- | --- | --- | --- | --- | --- | --- |
| **Inhibition of SARS-CoV-2 fusion** | | | | | | |
| Receptor-binding domain of the S1 Spike Protein | REGN3051 and REGN3048 MAbs,  Regdanvimab,  Bamlanivimab Estesevimab, Casirivimab, Imdevimab | S1 Spike Protein targeted antibodies | Exhibited efficacy *in vitro* | NCT03301090  NCT04602000  NCT04427501  NCT04452318 | Narrow therapeutic range | [51] |
| S2 subunit of S Protein | H2RP and P1 peptides | Prevent the fusion of virus with host cell | Peptide anti-HIV has been advertised | NCT0454684,  NCT04627233 | Their biodistribution would be limited to the upper airways (nasal and oral cavity) | [52,53] |
| TMPRSS2 | Camostat Mesylate  Nafamostat  Aprotinin | Virus entry pathway inhibitor | *In-vitro* it is having positive results. Reduction in lung cell line infection. | NCT04608266  NCT04418128  NCT04527133 | Adverse effects such as pruritis,  headache etc. | [54] |
| S Protein Trimerization | Arbidol | Inhibits the viral envelope's membrane fusion | Binding to HA protein | NCT04350684 | Narrow therapeutic range | [55,56] |
| **Inhibition of endocytosis** | | | | | | |
| Endosomal acidification | Chloroquine  Hydroxychloroquine | Multiple pathways impede viral entry and endocytosis and host immunomodulatory effects | Broad spectrum, SARS-CoV-2 patient shows good recovery | NCT04303507  NCT04860284 | Not enough available clinical evidence to indicate efficacy. Data is controversial | [57,58] |
| Clathrin mediated endocytosis | Oubain | ATP1A1- binding steroids; inhibiting endocytosis mediated by clathrin | Active against MERS-CoV | - | cardiac toxicity | [57] |
| **Inhibition of viral enzyme** | | | | | | |
| 3CLpro | Lopinavir | Inhibition of 3-chymotrypsin like protease activity | Broad spectrum | NCT04455958 | Toxicity | [59] |
| PLpro | GRL0617 | Inhibits Papain like protease activity | Narrow spectrum | - | Clinical data  is not available | [60] |
| RdRp | Remdesivir  Favipiravir  Ribavirin | Viral-RNA synthesis inhibitor | At high dose it is effective. | NCT04292730  NCT04694612  NCT04828564 | Common Side effects are seen, and high doses can be serious. | [61] |
| **Inhibition of viral envelope (E), nucleocapsid (N), membrane (M), and accessory proteins** | | | | | | |
| E and M protein | siRNA | The short dsRNA chains  Interfering with the  SARS-CoV expression  Proteins | Shows successful in vitro research results | - | Ideal conveyance technique in people is unsure | [62] |
| N protein | PJ34 | Deteriorates viral replication | Efficient in *in vitro* experiments with animals | - | Ideal conveyance technique  in people is unsure | [62] |
| Membrane and accessory proteins | LJ001 and JL103 | Membrane induced  damage | Therapeutic range is good as antiviral agent | - | Effectiveness to be established for COVID-19 | [62] |
| **Blocking cytokine storm** | | | | | | |
| TNF-α converting enzyme | Golimumab  adalimumab | Inhibits TNF-α | Good clinical and laboratory response | NCT00265083  NCT04705844 | expensive and yet there are no published trials with these drugs | [63] |
| IL-6 | Tocilizumab  Sarilumab | Inhibit IL-6 signalling and prevents IL-6 receptor activation | Showed good result against SARS-CoV-2 | NCT04320615  NCT04327388 | Adverse effects headache, diarrhoea and neutropenia are seen | [64] |
| JAK-STAT pathway | Baricitinib | Inhibits AAK1, the entry of SARS-CoV-2 into the host cells is prevented.  Inhibition of ACE2 development, which is dependent on JAK | Shows good clinical improvement | NCT04401579 | Common side effects are noted | [65] |
