# Supplementary material for: A global picture: therapeutic perspectives for COVID-19
Source: Immunotherapy. 2022 Feb 21:10.2217/imt-2021-0168. doi: 10.2217/imt-2021-0168 (PMC8884157; doi:10.2217/imt-2021-0168)
Supplement: Supplementary file 2 [file supplementary_table_2.docx]

**Supplementary Table 2:** Repurposed drugs currently practiced for COVID-19 management.

| **Drug** | **Therapeutic activity** | **Mechanism** | **Class** | **References** |
| --- | --- | --- | --- | --- |
| Remdesivir | Antiviral | It inhibits RNA polymerase, thereby inhibiting virus replication | Nucleotide analog prodrug | [75,76] |
| Chloroquine | Antiviral  Antimalarial | Prevents virus entry by glycosylation ACE2 | 9-aminoquinoline | [75–77] |
| Hydroxychloroquine | Antiviral  Antimalarial | Prevents endosomal fusion via pH increase | Chloroquine derivative | [75,77,78] |
| Camostat  mesylate | Antiviral | Prevents entry of the virus into a host cell | TMPRSS2 activity inhibitor | [76,79] |
| Azithromycin | Antibiotic  Immunomodulatory  Antiviral | Change in pH that leads to damage to trans Golgi network and lysosomes additionally causes glycosylation | Macrolide family | [80–83] |
| Lopinavir/  ritonavir | Antiviral | Inhibits replication of virus by blocking the protease enzyme of the virus | HIV-1 protease inhibitor | [84] |
| Arbidol | Antiviral | Prevents entry of the virus into a host cell, promotes interferon production, causes macrophage activation | Indole derivative | [85] |
| Dexamethasone | anti-inflammatory | Reduces the increase of cytokines caused by a virus | Glucocorticosteroid | [85–87] |
| Tocilizumab | Biological agent | Decreases cytokine storm caused by a virus | Recombinant monoclonal immunoglobulin (IgG1)antibody | [79,85,88] |
| Favipiravir (Fabiflu) | Anti-viral | It serves as a substrate for the enzyme RNA-dependent RNA-polymerase (RdRp) | Nucleotide analog prodrug | [42,89,90] |
| Nafamostat | Antiviral | Prevents entry of virus in a host cell, inhibits membrane fusion | Transmembrane protease serine 2 inhibitor  (TMPRSS2) | [42,91] |
| Ribavirin | Antiviral | Inhibiting viral replication by targeting Inosine monophosphate dehydrogenase | Inosine monophosphate dehydrogenase inhibitor | [92] |
| Ivermectin | Antiviral | Initial studies showed that it lowers the transport of viral RNA, however the data is controversial with many publications being retracted | Antiparasitic drug | [93] |
