# Supplementary material for: A global picture: therapeutic perspectives for COVID-19
Source: Immunotherapy. 2022 Feb 21:10.2217/imt-2021-0168. doi: 10.2217/imt-2021-0168 (PMC8884157; doi:10.2217/imt-2021-0168)
Supplement: Supplementary file 3 [file supplementary_table_3.docx]

**Supplementary Table 3.** Summary of some COVID-19 vaccines at different clinical stages of development

| **Type of Vaccine** | **Name of vaccine** | **Developer** | **Clinical stage** | **Clinical Trial Number** | **Timing of doses** |
| --- | --- | --- | --- | --- | --- |
| Inactivated virus vaccines | Inactivated (Vero Cells) | Chinese Academy of Medical Sciences | Phase 3 | NCT04412538  NCT04470609  NCT04412538  NCT04659239 | Days 0, 28 |
|  | Inactivated (Vero cell) | Sinopharm+China Biotec Group + Wuhan Institute of Biological Products | Phase 3 | NCT04510207  [NCT04612972](https://clinicaltrials.gov/ct2/show/NCT04612972) | Days 0, 21 |
|  | BBIBP-CorV | Sinopharm+China Biotec Group + Beijing Institute of Biological Products | Phase 3 | [NCT04560881](https://clinicaltrials.gov/ct2/show/NCT04560881?term=vaccine&cond=covid-19&draw=2&rank=3) | Days 0, 21 |
|  | Inactivated | Shifa Pharmed Industrial Co | Phase 3 | [IRCT20201202049567N1](https://covid19.trackvaccines.org/vaccines/83/#trial-irct20201202049567n1)  [IRCT20201202049567N3](https://covid19.trackvaccines.org/vaccines/83/#trial-irct20201202049567n3)  [IRCT20201202049567N3](https://covid19.trackvaccines.org/vaccines/83/#trial-irct20201202049567n3) | Days 0, 28 |
|  | Inactivated | Valneva VLA2001 | Phase 3 | [NCT04671017](https://covid19.trackvaccines.org/vaccines/69/#trial-nct04671017)  [NCT04671017](https://covid19.trackvaccines.org/vaccines/69/#trial-nct04671017)  [NCT04864561](https://covid19.trackvaccines.org/vaccines/69/#trial-nct04864561) | Days 0, 28 |
|  | Inactivated | The Scientific and Technological Research Council of Turkey | Phase 1 | [NCT04866069](https://covid19.trackvaccines.org/vaccines/112/#trial-nct04866069) | Days 0, 28 |
|  | KD-414 | KM Biologics Co Ltd | Phase 2 | [jRCT2071200106](https://covid19.trackvaccines.org/vaccines/115/#trial-jrct2071200106) [jRCT2071200106](https://covid19.trackvaccines.org/vaccines/115/#trial-jrct2071200106) | Days 0, 28 |
| Viral vector (non-replicating) vaccines | COVIVAC | Institute of Vaccines and Medical Biologicals | Phase 2 | [NCT04830800](https://covid19.trackvaccines.org/vaccines/107/#trial-nct04830800)  [NCT04830800](https://covid19.trackvaccines.org/vaccines/107/#trial-nct04830800) | Days 0, 28 |
|  | GRAd-COV2 | ReiThera | Phase 3 | [NCT04791423](https://covid19.trackvaccines.org/vaccines/13/#trial-nct04791423)  [NCT04528641](https://covid19.trackvaccines.org/vaccines/13/#trial-nct04528641)  [NCT04791423](https://covid19.trackvaccines.org/vaccines/13/#trial-nct04791423) | Days 0, 21 |
|  | LV-SMENP | Shenzen Geno-Immuno Medical Institute | Phase 2 | [NCT04276896](https://covid19.trackvaccines.org/vaccines/62/#trial-nct04276896)  [NCT04276896](https://covid19.trackvaccines.org/vaccines/62/#trial-nct04276896) | Days 0, 28 |
|  | hAd5-Covid-19 | ImmunityBio Inc | Phase 2 | [NCT04843722](https://covid19.trackvaccines.org/vaccines/49/#trial-nct04843722)  [NCT04845191](https://covid19.trackvaccines.org/vaccines/49/#trial-nct04845191) | Days 0, 28 |
| Protein subunit vaccine | Recombinant SARS-CoV-2 vaccine (CHO Cell) | Anhui Zhifei Longcom Biopharmaceutical + Institute of Microbiology, Chinese Academy of Sciences | Phase 3 | [NCT04646590](https://clinicaltrials.gov/ct2/show/NCT04646590) | Days 0, 28; or Days 0, 28, 56 |
|  | Recombinant Sf9 cell | West China Hospital | Phase 2 | [NCT04718467](https://covid19.trackvaccines.org/vaccines/33/#trial-nct04718467) | Days 0, 14, 28 |
|  | Nanocovax | Nanaogen | Phase 2 | [NCT04683484](https://covid19.trackvaccines.org/vaccines/72/#trial-nct04683484)  [NCT04683484](https://covid19.trackvaccines.org/vaccines/72/#trial-nct04683484) | Days 0, 28 |
|  | Recombinant trimeric subunit S protein vaccine [133] | Clover Biopharmaceuticals Inc. | Phase 1 | N/A | Days 0, 28 |
|  | CoVac-1 | Tuebingen | Phase 1 | [NCT04546841](https://covid19.trackvaccines.org/vaccines/41/#trial-nct04546841) | Days 0, 28 |
| RNA based vaccines [18] | CVnCoV Vaccine | CureVac AG | Phase 3 | [NCT04674189](https://www.clinicaltrials.gov/ct2/show/NCT04674189?id=NCT04639466+OR+NCT04655625+OR+NCT04662697+OR+NCT04683224+OR+NCT04668339+OR+NCT04674189+OR+NCT04665258+OR+NCT04646590+OR+NCT04642638+OR+NCT04656613+OR+NCT04648800+OR+NCT04649515+OR+NCT04677660+OR+NCT04668625+OR+NCT04649021+OR+NCT04649151+OR+NCT04659486+OR+NCT04664075&draw=2&rank=3&load=cart)  [NCT04838847](https://clinicaltrials.gov/ct2/show/NCT04838847)  [NCT04860258](https://clinicaltrials.gov/ct2/show/NCT04860258?term=NCT04860258&draw=2&rank=1) | Days 0, 21 |
|  | mRNA | Walvax | Phase 3 | [NCT04847102](https://covid19.trackvaccines.org/vaccines/23/#trial-nct04847102) | Days 0, 28 |
|  | BNT162b1 | Pfizer/BioNTech | Phase 4 | [NCT04368728](https://covid19.trackvaccines.org/vaccines/38/#trial-nct04368728)  [NCT04368728](https://covid19.trackvaccines.org/vaccines/38/#trial-nct04368728)  [ChiCTR2000034825, NCT04523571](https://covid19.trackvaccines.org/vaccines/38/#trial-chictr2000034825-nct04523571) | Days 0, 28 |
|  | MRT5500 | Sanofi Pasteur | Phase 2 | [NCT04798027](https://covid19.trackvaccines.org/vaccines/99/#trial-nct04798027)  [NCT04798027](https://covid19.trackvaccines.org/vaccines/99/#trial-nct04798027) | Days 0, 21 |
|  | DS-5670a | Daiichi Sankyo Co Ltd | Phase 2 | [NCT04821674](https://covid19.trackvaccines.org/vaccines/105/#trial-nct04821674)  [NCT04821674](https://covid19.trackvaccines.org/vaccines/105/#trial-nct04821674) | Days 0, 14 |
|  | LNP-nCoVsaRNA | Imperial College London [134] | Phase 1 | N/A | N/A |
| DNA based vaccines[135] | DNA plasmid vaccine | *In vivo* pharmaceuticals international vaccine institute [136] | Phase 1/2 | N/A | Days 0, 28 |
|  | DNA plasmid vaccine+ Adjuvant | Osaka university/AnGes/Takaar | Phase 1/2 | N/A | Days 0, 14 |
|  | INO-4800 | Inovio | Phase 3 | [NCT04336410](https://covid19.trackvaccines.org/vaccines/17/#trial-nct04336410)  [NCT04447781](https://covid19.trackvaccines.org/vaccines/17/#trial-nct04447781)  [NCT04642638](https://covid19.trackvaccines.org/vaccines/17/#trial-nct04642638) | Days 0, 28 |
|  | Covigenix VAX-001 | Entos Pharmaceuticals Inc | Phase 2 | [NCT04591184](https://covid19.trackvaccines.org/vaccines/64/#trial-nct04591184)  [NCT04591184](https://covid19.trackvaccines.org/vaccines/64/#trial-nct04591184) | Days 0, 28 |
|  | GX-19 | Genexine Consortium | Phase 1/2 | N/A | Days 0, 28 |
