# Supplementary material for: A global picture: therapeutic perspectives for COVID-19
Source: Immunotherapy. 2022 Feb 21:10.2217/imt-2021-0168. doi: 10.2217/imt-2021-0168 (PMC8884157; doi:10.2217/imt-2021-0168)
Supplement: Supplementary file 4 [file supplementary_table_4.docx]

**Supplementary Table 4.** Summary of monoclonal antibody therapeutics for COVID-19 at different clinical stages of development

| **Phase** | **Monoclonal antibody** | **Company** | **Mechanism of action** | **Ref.** |
| --- | --- | --- | --- | --- |
| Pre-clinical phase | S309 | Vir Biotechnology | SARS-CoV-2 neutralization *via* the S receptor binding domain (RBD) | [147] |
|  | P2C-1F11 | ACRO Biosystems | Resembles ACE2 in binding to RBD site of SARS-CoV-2 | [148] |
|  | B38 | Absolute Antibody | Binds to S-protein, preventing the virus from attaching to the host cell (spike inhibition) | [149] |
|  | 2-15 | Innovation Gillingham | Binds to S-protein and inhibits the binding of the virus to the host cell (spike inhibition) | [150] |
|  | CB6 | Junshi Biosciences | Binds to S-protein and inhibits the binding of the virus to the host cell (spike inhibition) | [151] |
|  | CC12.1 | Geneva Antibody facility | ACE2 inhibitory effect | [152] |
|  | BD-368-2 | Research team at Peking University | ACE2 inhibitory effect | [153] |
| Phase 1 | Anti-Spike (S) SARS-CoV-2 monoclonal mntibodies | Regeneron Pharmaceuticals | Spike inhibition | [154] |
| Phase 2 | Gimsilumab | Roivant’s | Anti-IL-6, IL-23, and tumor necrosis factor-alpha | [155] |
|  | Leronlimab | CytoDyn | CCR5 inhibition | [88] |
|  | Sarilumab | Regeneron Pharmaceuticals + Sanofi | IL-6 inhibitor | [156] |
|  | Canakinumab | Novartis | Anti-IL-1β | [157] |
| Phase 3 | Lenzilumab | Creative Biolabs | GM-CSF inhibitor | [158] |
|  | Emapalumab | Gamifant | Antibody against interferon-gamma | [159] |
| Phase 4 | Tocilizumab | Roche | IL-6 inhibitor | [159] |
