# Supplementary material for: A global picture: therapeutic perspectives for COVID-19
Source: Immunotherapy. 2022 Feb 21:10.2217/imt-2021-0168. doi: 10.2217/imt-2021-0168 (PMC8884157; doi:10.2217/imt-2021-0168)
Supplement: Supplementary file 5 [file supplementary_table_5.docx]

**Supplementary Table 5.** Phytochemicals that may be beneficial as adjunct agents in COVID-19

| **Plant** | **Phytochemical** | **Mechanism** | **Reference** |
| --- | --- | --- | --- |
| *Glycyrrhiza glabra;*  *Glycyrrhiza uralensis*  (liquorice) | Glycyrrhizin | Liquorice has shown inhibition of viral replication. Adsorption/penetration of virus by host cell is inhibited.  Drug induced nitrous oxide  Synthase is showing good viral inhibition activity. | [166] |
| *Bupleurum spp.;*  *Heteromorpha spp.;*  *Scrophularia scordonia* | Saikosaponin B2 | Viral replication at early stage is interfered. | [167] |
| *Stephania tetrandra* | TET  FAN  CEP | By targeting S and N protein it inhibits viral replication and it’s  Entry. | [168] |
| *Isatis indigotica* | Indigo  Sinigrin  Hesperetin | Target on 3CLpro protease and nsP13 helicase. | [169] |
| *Torreya nucifera* | Amentoflavone | It noncompetitively blocks and  Cleave 3CLpro of SARS-CoV in this way viral replication is hindered. | [170] |
| *Linum usitatissimum Linn.* | Herbacetin | Cleave 3CLpro of SARS-CoV and hinder the viral replication *via* blocking it. | [171] |
| *Rhus succedanea* | Rhoifolin | Cleave 3CLpro of SARS-CoV and hinder the viral replication *via* blocking it. | [172] |
| *Cirsium chanreoenium* | Pectolinarin | Cleave 3CLpro of SARS-CoV and hinder the viral replication *via* blocking it. | [173] |
| *Houttuynia cordata* | Houttuynia cordata Thunb water extract | Blocks viral replication by an  effect on 3CLpro and *via*  immunostimulatory effects | [174] |
| *Rheum palmatum L.*  *(Chinese rhubarb)* | Rheum palmatum L. extracts | Restricts the interaction S protein and ACE2 and Cleave 3CLpro of SARS-CoV and hinder the viral replication *via* blocking it. | [175] |
| *Cibotium barometz* | Rhizoma cibotii | Blocks viral cytopathogenic  effect and restricts viral  Replication by targeting 3CLpro. | [176,177] |
| *Dioscorea batatas* | Discoreae rhizome | Blocks viral cytopathogenic  effect and restricts viral  replication by targeting 3CLpro. | [176] |
| *Ceratonia siliqua* | Myricetin | Restricts viral replication by  blocking the ATPase activity of helicase protein  nsP14; also shown antioxidant property. | [178] |
| *Galla chinensis* | TGG | Hinders with viral cell fusion  By targeting to the S protein. | [179] |
