# Supplementary material for: A global picture: therapeutic perspectives for COVID-19
Source: Immunotherapy. 2022 Feb 21:10.2217/imt-2021-0168. doi: 10.2217/imt-2021-0168 (PMC8884157; doi:10.2217/imt-2021-0168)
Supplement: Supplementary file 6 [file supplementary_table_6.docx]

**Supplementary Table 6:** Vitamins and amino acid supplements against COVID-19

| **Supplements** | **Sources** | **Active constituents** | **Clinical Trials** | **References** |
| --- | --- | --- | --- | --- |
| N-acetyl cysteine | A derivative of the cysteine amino acid. | N-acetyl cysteine | NCT04419025 | [190,191] |
| L-Glutamine | Cruciferous vegetables, or the sulfur-rich foods, like garlic or onion. | L-Glutamine | NCT04909905 | [192] |
| Combined metabolic activators (CMAs) | Glutathione and NAD+ precursors | L-serine, N-acetyl-l-cysteine, nicotinamide riboside, and l-carnitine tartrate, salt form of l-carnitine | Open‐label phase‐2 and double‐blinded phase‐3 clinical trials | [191] |
| Vitamin C | Derived from fruits and vegetables of the citrus family, and includes tomatoes, potatoes, peppers, kiwifruit, broccoli, strawberries, and cantaloupe. | Ascorbic acid  Citric acid | NCT04530539,  NCT04357782,  NCT04664010 | [193] |
| Vitamin D | The primary ingredient is found in fatty fish (such as tuna fish and salmon fish) and their liver oils. It is also obtained from cheese, cow liver oil, and egg yolks in the form of vitamin D1, D2, D3, D4, and D5. | Ergocalciferol  Cholecalciferol  22-dihydroergocalciferol Sitocalciferol  Ergocalciferol with lumisterol | NCT04487951,  NCT04709744,  NCT04536298,  NCT04449718,  NCT04868903, | [194,195] |
| Vitamin K | Vegetables such as Brussels sprouts, broccoli, cauliflower, and cabbage | Vitamin K2 in the form of Menaquinone-7 (MK-7) | NCT04770740 | [186] |
| Vitamin A | Yellow, red and green (leafy) vegetables | Retinoic acid (RA) | NCT04920760 | [196] |
